# Targeting cancer hallmarks with microbiome-derived bacteriocins and postbiotics: molecular mechanisms and translational opportunities in oncology

**DOI:** 10.3389/fonc.2026.1899156

**Published:** 2026-09-03

**Authors:** Radhamanalan Guhanraj

**Affiliations:** Department of Microbiology, Saveetha Dental College, Chennai, Tamil Nadu, India

**Keywords:** artificial intelligence, bacteriocins, cancer therapeutics, microbiome-derived, metabolites, postbiotics, precision oncology

## Abstract

**Background:**

The human microbiome is an abundant reservoir of bioactive molecules with considerable therapeutic potential in oncology. Among these microbial products, bacteriocins and other postbiotic metabolites have emerged as promising anticancer agents due to their diverse biological activities and ability to modulate multiple cancer-related pathways. Growing evidence suggests that microbiome-derived compounds may provide novel opportunities for the development of safer and more targeted cancer therapeutics.

**Objectives:**

This review aims to comprehensively evaluate the current knowledge on bacteriocins and postbiotic metabolites as anticancer agents, highlighting their sources, structural and functional properties, mechanisms of action, computational-assisted discovery approaches, nanotechnology-based delivery systems, and challenges associated with clinical translation.

**Methods:**

A critical analysis of the available literature was conducted to summarize the biological characteristics of bacteriocin-producing microorganisms, the classification and mechanisms of bacteriocins, and the anticancer activities of major postbiotic metabolites. Recent advances in molecular docking, molecular dynamics simulations, systems biology, artificial intelligence, and nanotechnology-based drug delivery platforms were also examined to assess their contributions to therapeutic development.

**Results:**

Bacteriocins exhibit anticancer effects through multiple mechanisms, including disruption of cancer cell membranes, induction of apoptosis, regulation of oxidative stress, inhibition of cell proliferation, suppression of angiogenesis and metastasis, epigenetic modulation, and enhancement of antitumor immune responses. In addition, postbiotic metabolites such as short-chain fatty acids, indole derivatives, reuterin, and microbial exopolysaccharides contribute to tumour suppression and modulation of host–microbe interactions. Computational approaches have accelerated the identification and optimization of bacteriocin-derived therapeutic candidates, while nanotechnology-based delivery systems have improved their stability, bioavailability, and tumour-targeting capabilities. Nevertheless, challenges including limited clinical evidence, non-standardized experimental methodologies, poor pharmacokinetic properties, manufacturing constraints, and regulatory barriers remain significant obstacles to clinical implementation.

**Conclusion:**

Bacteriocins and postbiotic metabolites represent a versatile and promising class of microbiome-derived anticancer agents with substantial potential for precision oncology. The integration of microbiome science, synthetic biology, computational drug discovery, and advanced drug delivery technologies is expected to facilitate the translation of these natural compounds into clinically effective cancer therapeutics. Future research should focus on mechanistic validation, large-scale production, pharmacological optimization, and well-designed clinical studies to accelerate their development and therapeutic application.

## Graphical Abstract

**Figure:**
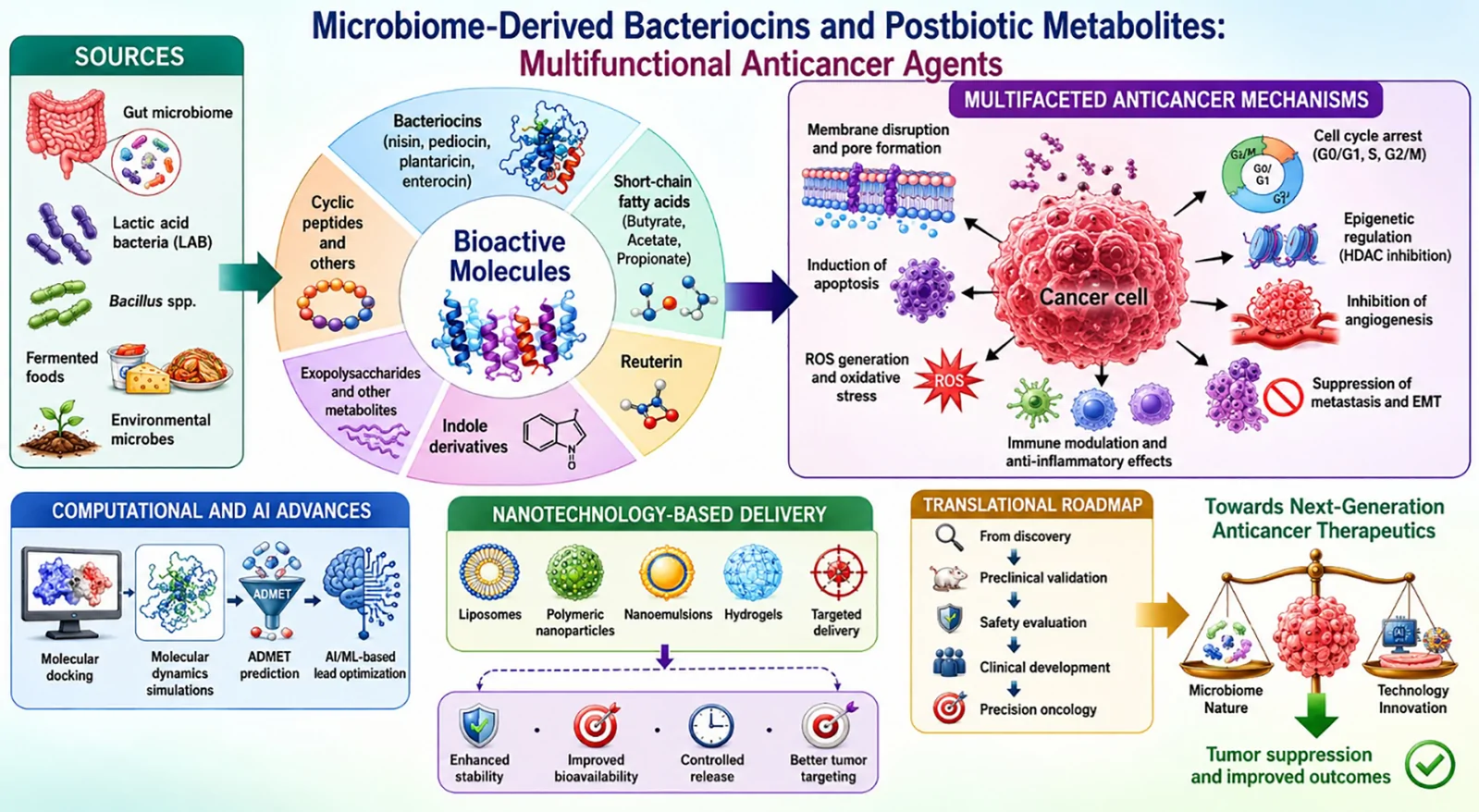

## Introduction

Cancer remains one of the most significant global public health challenges and is a leading cause of morbidity and mortality worldwide (1, 2). Recent global estimates indicate that millions of new cancer cases are diagnosed annually, with incidence projected to increase substantially over the coming decades because of population growth, population ageing, urbanization, environmental exposures, and lifestyle-related risk factors (3, 4). Lung, breast, colorectal, prostate, liver, and gastric cancers account for a large proportion of cancer-related deaths worldwide (3–5). The burden of cancer extends beyond healthcare expenditures and includes productivity loss, psychological distress, reduced quality of life, and long-term disability (6, 7). Despite major advances in cancer prevention, diagnosis, and treatment, many malignancies continue to have poor prognoses, particularly when diagnosed at advanced stages (8). Poor therapeutic responses and disease recurrence are largely attributed to tumour heterogeneity, genetic instability, and complex interactions within the tumour microenvironment (9, 10). Consequently, the development of novel therapeutic strategies remains a major priority in contemporary oncology research (10).

Chemotherapy and targeted therapies have significantly improved cancer management; however, they remain associated with several limitations (11, 12). Conventional chemotherapy has long served as a cornerstone of cancer treatment (11). Cytotoxic agents, including platinum-based compounds, anthracyclines, antimetabolites, and alkylating agents, exert their anticancer effects by interfering with DNA synthesis, cell division, and other essential cellular processes involved in tumour growth and survival (12). Although these therapies have improved survival outcomes across multiple cancer types, their lack of specificity frequently results in substantial toxicity to normal tissues, leading to adverse effects such as myelosuppression, gastrointestinal dysfunction, cardiotoxicity, nephrotoxicity, and neurotoxicity (13).

The emergence of targeted therapies represented a major advancement in precision oncology (14). These agents selectively inhibit molecular targets involved in tumour initiation, progression, and metastasis, including epidermal growth factor receptors, vascular endothelial growth factor signalling pathways, cyclin-dependent kinases, and oncogenic kinases (14, 15). While targeted therapies are generally more selective and associated with lower systemic toxicity than conventional chemotherapy, their clinical benefits are often limited by intrinsic or acquired resistance mechanisms (15, 16). Furthermore, the effectiveness of many targeted agents depends on the presence of specific molecular or genetic alterations, restricting their use to selected patient populations (16). Additionally, the high cost of targeted therapeutics, the risk of adverse effects associated with prolonged treatment, and the occurrence of non-responsiveness among certain patients underscore the urgent need for innovative and alternative strategies for cancer drug discovery and development (17).

## Drug resistance in cancer

One of the greatest challenges in cancer treatment is the development of drug resistance (18). Resistance may be either intrinsic, existing before treatment begins, or acquired, emerging during the course of therapy, ultimately contributing to disease progression and treatment failure (18, 19). Several biological mechanisms underlie this phenomenon. Cancer cells frequently acquire genetic mutations that alter drug targets, thereby reducing therapeutic efficacy (19). In addition, the upregulation of efflux transporters, particularly ATP-binding cassette (ABC) proteins, decreases intracellular drug accumulation and diminishes the cytotoxic effects of anticancer agents (20, 21). Furthermore, activation of compensatory signalling pathways enables tumour cells to bypass inhibited molecular targets and sustain their proliferative capacity (20).

The tumour microenvironment (TME) also plays a critical role in the development of therapeutic resistance (22). Interactions among tumour cells, stromal cells, immune cells, extracellular matrix components, and resident microorganisms create a protective niche that promotes tumour survival, adaptation, and disease progression (22, 23). Factors such as hypoxia, metabolic reprogramming, epigenetic alterations, and the presence of cancer stem cells further contribute to resistance against chemotherapy, radiotherapy, and targeted therapies (19, 22, 23). Given the multifactorial nature of drug resistance, there is increasing interest in identifying novel bioactive molecules with unique mechanisms of action that can overcome established resistance pathways or act synergistically with existing anticancer therapies (24, 25).

## Natural-product-based anticancer discovery

Natural products have long served as a valuable source of therapeutic agents and continue to inspire modern drug discovery efforts (26). A substantial proportion of clinically approved anticancer drugs are derived directly or indirectly from natural sources, including plants, microorganisms, marine organisms, and fungi (26, 27). Owing to their remarkable structural diversity and biological complexity, natural products provide unique chemical scaffolds that are often difficult to reproduce through conventional synthetic chemistry (27). Plant-derived compounds such as paclitaxel, vincristine, vinblastine, and camptothecin derivatives have demonstrated significant clinical efficacy in the treatment of various cancers (28).

Similarly, microbial metabolites have played a pivotal role in pharmaceutical development, leading to the discovery of compounds with antibacterial, antifungal, immunosuppressive, and anticancer activities (29). Recent advances in genomics, metabolomics, and systems biology have greatly expanded access to previously unexplored microbial resources, facilitating the identification of novel bioactive compounds (30). Microbial secondary metabolites can influence host physiology and mediate complex interspecies interactions, often exhibiting diverse biological activities (31). Many of these molecules possess antiproliferative, pro-apoptotic, anti-inflammatory, immunomodulatory, and antimetastatic properties, highlighting their potential as promising candidates for cancer therapy (10, 29, 30). Consequently, there is growing interest in the discovery and development of natural product-derived small molecules that can simultaneously target multiple cancer-related pathways while exhibiting minimal systemic toxicity (31, 32).

## Interactions between microbes and cancer

The human microbiome has emerged as a critical regulator of health and disease, influencing immune function, metabolism, inflammation, and cancer development (33). Diverse microbial communities inhabit the gastrointestinal tract, oral cavity, skin, respiratory tract, and genitourinary system, where they contribute significantly to the maintenance of host physiological homeostasis (33). Increasing evidence suggests that microbial dysbiosis plays an important role in carcinogenesis through several mechanisms, including chronic inflammation, production of genotoxic metabolites, immune dysregulation, and alterations in host signalling pathways (34). Specific microbial species have been associated with an increased risk of colorectal, gastric, hepatic, pancreatic, and oral cancers (33, 34).

Conversely, beneficial microorganisms can exert protective effects through the production of bioactive metabolites that suppress inflammation, enhance immune surveillance, inhibit pathogen colonization, and regulate cellular proliferation (35). In addition to influencing cancer initiation and progression, the microbiome can modulate responses to chemotherapy, radiotherapy, immunotherapy, and targeted therapies (36). Variations in microbial composition may affect drug metabolism, therapeutic efficacy, and treatment-associated toxicity (37). Consequently, increasing attention has been directed toward understanding the molecular crosstalk between host cells and microbial communities in the context of cancer (38). This rapidly evolving field has opened new opportunities for the discovery and development of microbiome-derived bioactive molecules as potential anticancer therapeutics (35–38).

## Bacteriocins and postbiotics in oncology

Bacteriocins and postbiotics are two important classes of microbiome-derived bioactive compounds that have attracted considerable attention because of their diverse biological activities and favourable safety profiles (39, 40). Bacteriocins are ribosomally synthesized antimicrobial peptides produced by a wide range of bacterial species, particularly lactic acid bacteria (39). Although traditionally investigated for their antimicrobial and food-preservative properties, accumulating evidence indicates that certain bacteriocins also possess selective anticancer activities (40, 41). These peptides exert antitumour effects through multiple mechanisms, including membrane disruption, mitochondrial dysfunction, induction of apoptosis, cell-cycle arrest, modulation of oxidative stress, and inhibition of signalling pathways involved in tumour growth and progression (41, 42). Notably, several bacteriocins have demonstrated preferential cytotoxicity toward malignant cells while exerting minimal effects on normal cells, highlighting their potential as targeted anticancer agents (**Table 1**) (43, 44).

**Table 1:** Anticancer potential of bacteriocin producing microorganisms.

Genus	Representative species	Major bacteriocins	Bacteriocin class	Biological properties	Advantages	Anticancer potential
*Lactobacillus*	*L. plantarum*, *L. acidophilus*, *L. rhamnosus*	Plantaricins, Acidocins, Salivaricins	Class I and II	Antimicrobial, immunomodulatory, probiotic-associated	GRAS status, gut compatibility, safety	Moderate to high
*Lactococcus*	*L. lactis*	Nisin, Lacticins	Primarily Class I	Strong antimicrobial and membrane-targeting activity	Extensive characterization, regulatory acceptance	High
*Leuconostoc*	*L. mesenteroides*, *L. gelidum*	Leucocins	Class II	Antimicrobial and preservative properties	High stability, food-grade source	Emerging
*Pediococcus*	*P. acidilactici*, *P. pentosaceus*	Pediocin PA-1	Class IIa	Potent membrane disruption	Thermal and pH stability	Moderate
*Enterococcus*	*E. faecium*, *E. faecalis*	Enterocins	Class II and III	Broad-spectrum antimicrobial activity	High potency and environmental tolerance	Moderate to high
*Bacillus*	*B. subtilis*, *B. coagulans*	Subtilin, Coagulin, Megacin	Class I and III	Highly stable and diverse mechanisms	High production yield, industrial scalability	High

**Figure 1:**
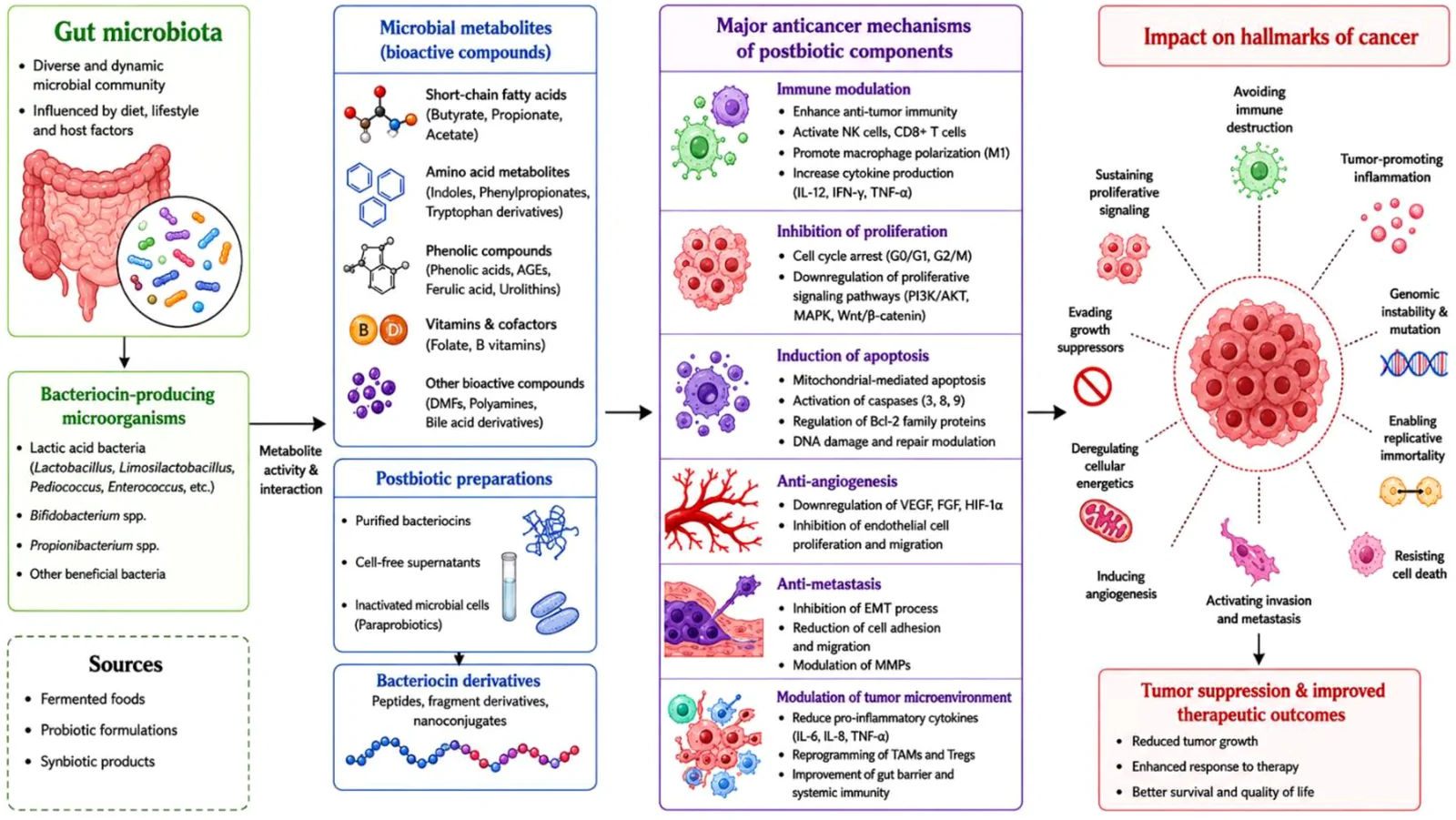
Microbiome-derived postbiotics and bacteriocins: sources, bioactive metabolites, and mechanisms of anticancer activity.

Postbiotics comprise a diverse group of non-viable microbial cells, cell components, and metabolites, including short-chain fatty acids, organic acids, enzymes, extracellular polysaccharides, cell wall constituents, peptides, and signalling molecules (**Figure 1**) (45). Compared with live probiotics, postbiotics offer several advantages, including enhanced stability, easier standardisation of production processes, extended shelf life, and improved safety by eliminating the risks associated with the administration of viable microorganisms (45, 46). Numerous postbiotic metabolites have been reported to exhibit anticancer properties through the regulation of apoptosis, autophagy, angiogenesis, immune responses, and epigenetic mechanisms (46–49). Furthermore, microbial metabolites provide valuable molecular scaffolds for the design and development of novel small-molecule inhibitors with improved pharmacological and therapeutic properties (49).

In light of the growing convergence of microbiome science, natural product chemistry, and cancer biology, bacteriocins and postbiotics have emerged as promising sources of innovative therapeutic candidates for oncology (50). In this context, computational drug discovery approaches, including molecular docking, virtual screening, molecular dynamics simulations, and structure-based drug design, have become indispensable tools for identifying and optimizing microbiome-derived compounds with anticancer potential (50–53).

## Computational drug discovery and scope

The conventional drug discovery process is often expensive, time-consuming, and associated with high attrition rates (54). The integration of computational methodologies has transformed this process by enabling the rapid identification, optimization, and evaluation of potential therapeutic candidates (54, 55). Modern computational drug discovery employs a wide range of approaches, including molecular docking, virtual screening, pharmacophore modelling, molecular dynamics (MD) simulations, quantitative structure–activity relationship (QSAR) analysis, machine learning, artificial intelligence (AI), network pharmacology, and systems biology (55, 56). These techniques facilitate the prediction of target–ligand interactions, binding affinities, pharmacokinetic properties, toxicity profiles, and biological pathways prior to experimental validation (56, 57).

Computational approaches are particularly valuable for microbiome-derived compounds, as they enable the efficient screening of large libraries of bacteriocins, peptides, and microbial metabolites against cancer-associated molecular targets (50, 54, 57). Such methods accelerate the identification of lead compounds capable of modulating key oncogenic processes, including cellular proliferation, apoptosis, metastasis, angiogenesis, immune evasion, and drug resistance (57, 58). Furthermore, advances in bioinformatics, structural biology, and artificial intelligence have facilitated the rational design of next-generation anticancer agents based on naturally occurring microbial molecules (55, 58, 59).

This review provides a comprehensive overview of the emerging role of bacteriocins, postbiotic metabolites, and microbiome-derived small molecules in anticancer drug discovery (25, 44). It examines current knowledge regarding the relationship between the microbiome and cancer, the biological properties of bacteriocins and postbiotics, and their underlying mechanisms of anticancer activity (37, 44, 58). Particular emphasis is placed on computational strategies for the identification, characterization, and optimization of microbiome-derived compounds for oncological applications (54, 55, 58). The review further highlights the application of molecular docking, virtual screening, molecular dynamics simulations, artificial intelligence-driven drug design, and network-based analyses in the discovery and development of novel anticancer therapeutics (55–60). In addition, critical challenges related to target specificity, bioavailability, toxicity, large-scale production, regulatory approval, and clinical translation are discussed (59, 60). Finally, future perspectives on microbiome-inspired precision oncology and the development of next-generation small-molecule anticancer agents are presented (58–60). By integrating advances in microbiology, computational biology, medicinal chemistry, and cancer therapeutics, this review highlights innovative strategies for the discovery and development of effective anticancer drugs derived from microbial bioactive compounds (25, 37, 44, 58–60).

## Gut microbiota and their derived bioactive metabolites in cancer

The gut microbiota is a complex and dynamic community of microorganisms that plays a fundamental role in maintaining host physiology by regulating metabolism, immune homeostasis, nutrient utilization, and intestinal barrier integrity (61). Increasing evidence indicates that alterations in the composition and function of the gut microbiota are closely associated with the initiation and progression of multiple cancer types (61, 62). Although changes in microbial communities have long been linked to disease, it is now recognized that many of their biological effects are mediated through microbiota-derived bioactive metabolites (62, 63). These small molecules function as key mediators of host–microbiome interactions by regulating intracellular signalling pathways involved in inflammation, immune surveillance, cell proliferation, apoptosis, angiogenesis, and genomic stability (63, 64). Consequently, microbial metabolites have emerged as promising candidates for cancer prevention and therapeutic intervention (64).

Among the best-characterized microbiota-derived metabolites are short-chain fatty acids (SCFAs), including acetate, propionate, and butyrate, which are produced through the bacterial fermentation of dietary fibre (65). SCFAs contribute to intestinal homeostasis by maintaining epithelial barrier integrity, serving as an energy source for colonocytes, and regulating immune responses (65). Among them, butyrate has attracted particular attention because of its ability to inhibit histone deacetylases (HDACs), thereby modulating gene expression through epigenetic mechanisms (66). This activity promotes cell-cycle arrest, induces apoptosis, suppresses inflammatory signalling, and restores the expression of tumour suppressor genes (66, 67). In addition, experimental studies have demonstrated that SCFAs enhance antitumor immunity by promoting regulatory immune cell function while reducing chronic inflammation, a key contributor to carcinogenesis (67).

Another important class of microbiota-derived bioactive molecules is bacteriocins, which are ribosomally synthesized antimicrobial peptides produced by several commensal bacterial species (68). Initially recognized for their antimicrobial activity, bacteriocins have recently gained attention for their selective anticancer properties (68, 69). Several bacteriocins exhibit preferential cytotoxicity toward malignant cells while exerting minimal effects on normal tissues (69). Their anticancer mechanisms include disruption of cancer cell membrane integrity, mitochondrial dysfunction, activation of intrinsic apoptotic pathways, modulation of intracellular calcium homeostasis, and inhibition of proliferative signalling pathways (69, 70). Representative bacteriocins, including nisin, pediocin, plantaricin, enterocin, and microcin, have demonstrated promising anticancer activity against colorectal, breast, oral, cervical, and head-and-neck cancer cell lines in experimental studies (68–70). These findings highlight the potential of bacteriocins as complementary therapeutic agents, either as standalone treatments or in combination with conventional anticancer therapies (69, 70).

Dietary tryptophan is metabolized by intestinal microorganisms into a variety of indole derivatives, including indole-3-acetic acid, indole-3-propionic acid, and indole-3-aldehyde (63, 65). These metabolites primarily regulate host physiology through activation of the aryl hydrocarbon receptor (AhR), which plays a critical role in immune regulation, epithelial barrier maintenance, and inflammatory signalling (63, 65). Balanced AhR activation contributes to tissue homeostasis and protection against inflammation-associated tumorigenesis (65). However, dysregulated AhR signalling may exert context-dependent effects that either suppress or promote cancer progression, underscoring the complexity of microbiome–host interactions (65, 66).

Gut microorganisms also convert primary bile acids into secondary bile acids, such as deoxycholic acid and lithocholic acid (66). These metabolites exert diverse biological effects depending on their concentration and physiological context (66). Certain secondary bile acids have been associated with oxidative stress, DNA damage, chronic inflammation, and activation of oncogenic signalling pathways, particularly in gastrointestinal malignancies (66, 67). In contrast, other bile acid metabolites exhibit anti-inflammatory and immunomodulatory activities, suggesting that microbial bile acid metabolism may either promote or inhibit tumour progression depending on the tumour microenvironment (67).

In addition to low-molecular-weight metabolites, many probiotic microorganisms produce exopolysaccharides (EPS) with immunomodulatory and anticancer properties (67, 68). These extracellular polymers regulate immune cell activation, reduce oxidative stress, inhibit tumour cell proliferation, and induce apoptotic signalling pathways (68). Their excellent biocompatibility and low toxicity have stimulated interest in their application as functional biomaterials and components of advanced drug delivery systems for cancer therapy (68, 69). Similarly, metabolites such as reuterin, conjugated linoleic acid (CLA), γ-aminobutyric acid (GABA), and microbial extracellular vesicles (EVs) are increasingly being investigated for their roles in regulating oxidative stress, immune responses, and intracellular signalling pathways associated with tumour progression (69, 70).

Emerging evidence also indicates that microbiota-derived metabolites influence the efficacy of cancer treatment (62, 69). Several studies have demonstrated that these metabolites modulate immune activation, inflammatory pathways, and drug metabolism, thereby affecting responses to chemotherapy, radiotherapy, and immunotherapy (62, 69, 70). For example, SCFAs have been shown to enhance cytotoxic CD8^+^ T-cell responses and improve the efficacy of immune checkpoint inhibitors in preclinical models (65, 67). These findings have stimulated growing interest in microbiome-based therapeutic strategies, including probiotics, prebiotics, synbiotics, engineered bacterial therapeutics, faecal microbiota transplantation (FMT), and purified postbiotic formulations aimed at improving therapeutic efficacy while minimizing treatment-related toxicity (62, 69, 70).

Despite these promising advances, several challenges continue to limit the clinical translation of microbiome-derived therapeutics (70). Most available evidence is derived from *in vitro* experiments and animal studies, whereas robust clinical trials remain limited (70). Interindividual variability in gut microbiota composition, differences in metabolite production, the lack of standardized postbiotic formulations, and an incomplete understanding of pharmacokinetic and pharmacodynamic properties represent major barriers to clinical implementation (70). Addressing these challenges will require standardized experimental protocols, multicentre clinical trials, and the integration of multi-omics technologies with computational biology to identify reliable biomarkers and therapeutic targets (61, 70).

Overall, gut microbiota-derived bioactive metabolites represent an emerging class of naturally occurring molecules with significant potential for cancer prevention and treatment (61–70). Their ability to regulate multiple hallmarks of cancer through coordinated effects on cellular signalling, immune function, and metabolism provides a strong biological foundation for future therapeutic development (63, 67, 69). Continued advances in precision medicine, artificial intelligence, microbiome engineering, and mechanistic research are expected to accelerate the translation of these microbial products into clinically relevant therapeutics for precision oncology (57, 59, 60, 70).

## Postbiotics and bacteriocin-derived small molecules

The growing interest in microbiome-based therapeutics has shifted attention beyond living microorganisms toward the bioactive compounds they produce (71). Among these compounds, postbiotics have emerged as a promising class of microbiome-derived products with considerable potential for biomedical and pharmaceutical applications (71, 72). Postbiotics are generally defined as preparations of inanimate microorganisms and/or their components that confer health benefits on the host (72, 73). They may also include a wide range of microbial metabolites and bioactive molecules produced during microbial growth and metabolism (71, 73). Unlike probiotic-based interventions, postbiotics do not contain viable microorganisms and therefore eliminate concerns related to microbial survival, colonization efficiency, horizontal gene transfer, and the risk of infection in immunocompromised individuals (65, 68, 72, 73).

Postbiotics encompass a diverse array of bioactive molecules, including short-chain fatty acids, organic acids, peptides, bacteriocins, enzymes, extracellular polysaccharides, cell wall fragments, biosurfactants, vitamins, and signalling molecules (71, 74). These compounds serve as important mediators of host–microbe communication and influence numerous physiological processes, including immune regulation, inflammatory responses, metabolic homeostasis, epithelial barrier integrity, and cellular signalling (74). In recent years, increasing evidence has highlighted the potential role of postbiotics in cancer prevention and therapy (74, 75). Many microbial metabolites exhibit antiproliferative, pro-apoptotic, anti-inflammatory, antioxidant, and immunomodulatory activities that can directly or indirectly influence tumour initiation, progression, and metastasis (75, 76). Furthermore, compared with live microbial formulations, postbiotics often demonstrate superior stability, easier standardization of production, extended shelf life, and improved safety profiles (72, 73, 76). These advantages make postbiotics attractive candidates for pharmaceutical development and therapeutic applications (76). Consequently, the growing integration of microbiome research with cancer biology has positioned postbiotics as an emerging source of innovative therapeutic molecules for oncology (74–76).

## Bacteriocin degradation products

**Figure 2:**
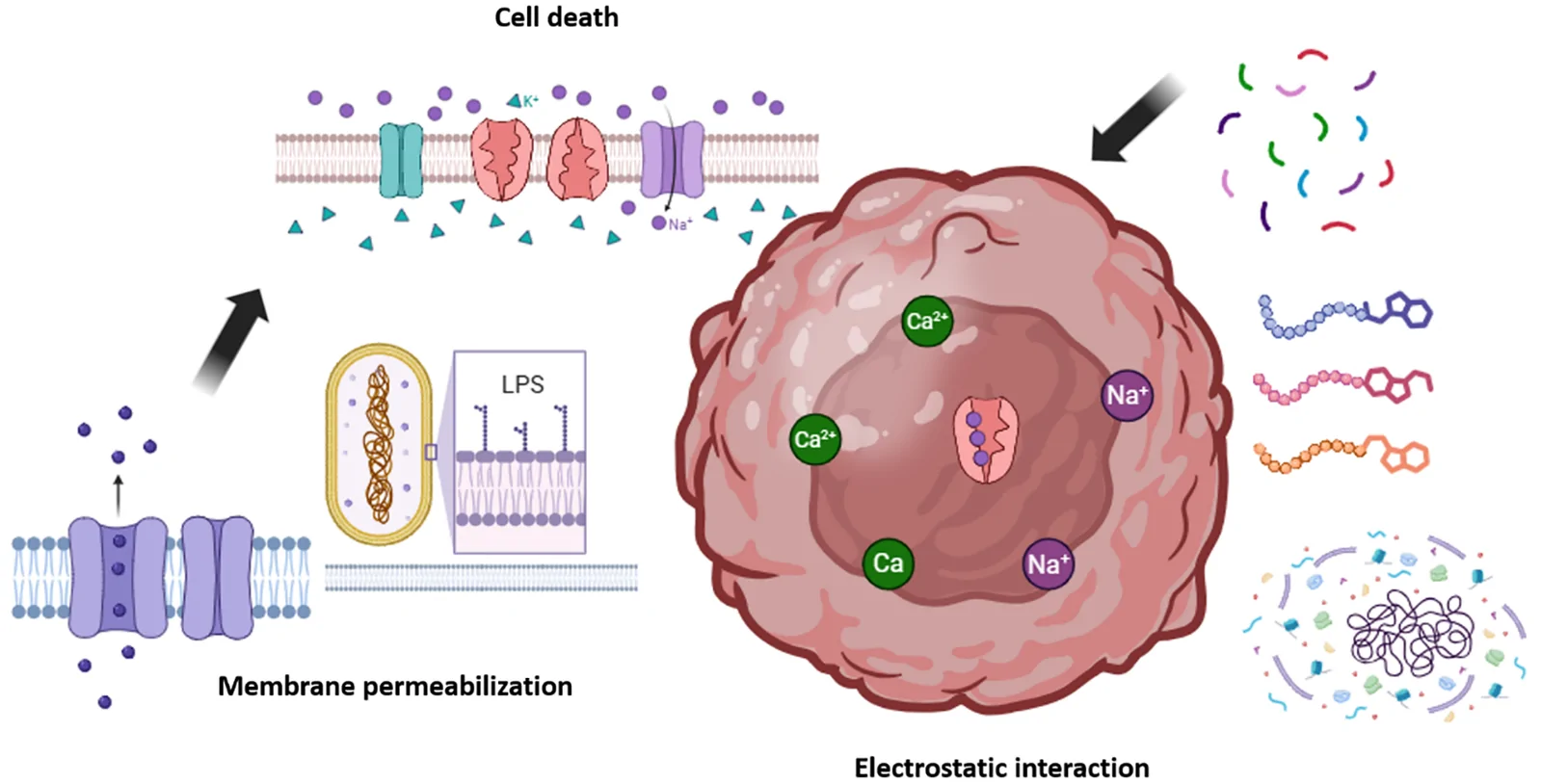
Mechanism of bacteriocin action on cancer cell membrane.

Although bacteriocins have traditionally attracted attention because of their antimicrobial and anticancer activities, growing evidence suggests that their degradation products may also possess significant biological functions (77). Bacteriocins can undergo enzymatic degradation, microbial metabolism, or environmental processing, resulting in the generation of smaller peptide fragments with distinct biological properties (77, 78). In some cases, these degradation products retain the biological activities of the parent molecule, whereas in others they exhibit enhanced functionality (78). Due to their smaller size, bacteriocin-derived fragments may demonstrate improved tissue penetration, enhanced bioavailability, and more favourable pharmacokinetic properties (78, 79). Proteolytic processing can generate bioactive peptides capable of modulating immune responses, influencing cellular signalling pathways, and interacting with biological membranes (79). Importantly, some bacteriocin-derived peptides have shown selective cytotoxicity toward cancer cells while exhibiting reduced toxicity toward normal tissues (**Figure 2**) (77, 79, 80). These observations have stimulated interest in the development of bacteriocin-inspired peptidomimetics and small-molecule analogues for therapeutic applications (77–79).

The biological activity of bacteriocin degradation products highlights the dynamic interactions that occur within the gastrointestinal tract and the tumour microenvironment (81). Understanding the metabolic fate of bacteriocins following administration is therefore essential for evaluating their pharmacological properties, therapeutic efficacy, and safety (81). Furthermore, characterization of these degradation pathways may facilitate the identification of novel bioactive molecules with improved stability and clinical utility (82). As a result, bacteriocin-derived fragments are increasingly being recognized as valuable leads for the development of next-generation antimicrobial and anticancer therapeutics (82).

## Biosynthetic pathways of postbiotic metabolites

The biosynthesis of postbiotic metabolites is governed by diverse microbial metabolic pathways that convert dietary substrates and endogenous compounds into biologically active molecules (83). These pathways are influenced by microbial composition, nutrient availability, environmental conditions, and host physiological status (83). One of the most important pathways involves the fermentation of dietary fibres and resistant starches by gut microorganisms, resulting in the production of short-chain fatty acids (SCFAs) such as acetate, propionate, and butyrate (83, 84). In addition, microbial amino acid metabolism contributes to the generation of indole derivatives, phenolic compounds, and numerous signalling molecules with important physiological functions (84). Certain bacterial species also synthesize extracellular polysaccharides, cyclic peptides, and other bioactive metabolites through specialized biosynthetic pathways (81, 83).

Recent advances in metabolomics, metagenomics, and systems biology have substantially improved our understanding of postbiotic biosynthesis (83, 85). The integration of computational biology with microbial metabolic network analysis has enabled the identification of key enzymes, regulatory pathways, and biosynthetic gene clusters involved in postbiotic production (84, 85). These approaches facilitate the discovery of novel metabolites and support the rational engineering of microbial strains capable of producing therapeutic compounds at industrial scales (84). As a result, microbial metabolites are increasingly recognized as a vast reservoir of structurally diverse bioactive molecules with significant potential for anticancer drug discovery (75, 84).

Unlike many conventional chemotherapeutic agents that primarily target a single molecular pathway, postbiotic metabolites often exhibit pleiotropic biological activities (75, 80). These compounds can modulate inflammation, immune responses, cellular metabolism, oxidative stress, angiogenesis, apoptosis, and epigenetic regulation (75, 80, 83). Furthermore, many postbiotic metabolites influence key cancer-related signalling pathways, including NF-κB, PI3K/Akt, Wnt/β-catenin, and MAPK, as well as interactions between tumour cells and the tumour microenvironment (79, 83, 84). Such multifunctional properties have generated considerable interest in their application as lead compounds for precision oncology and next-generation anticancer therapeutics (75, 84).

## Butyrate

**Figure 3:**
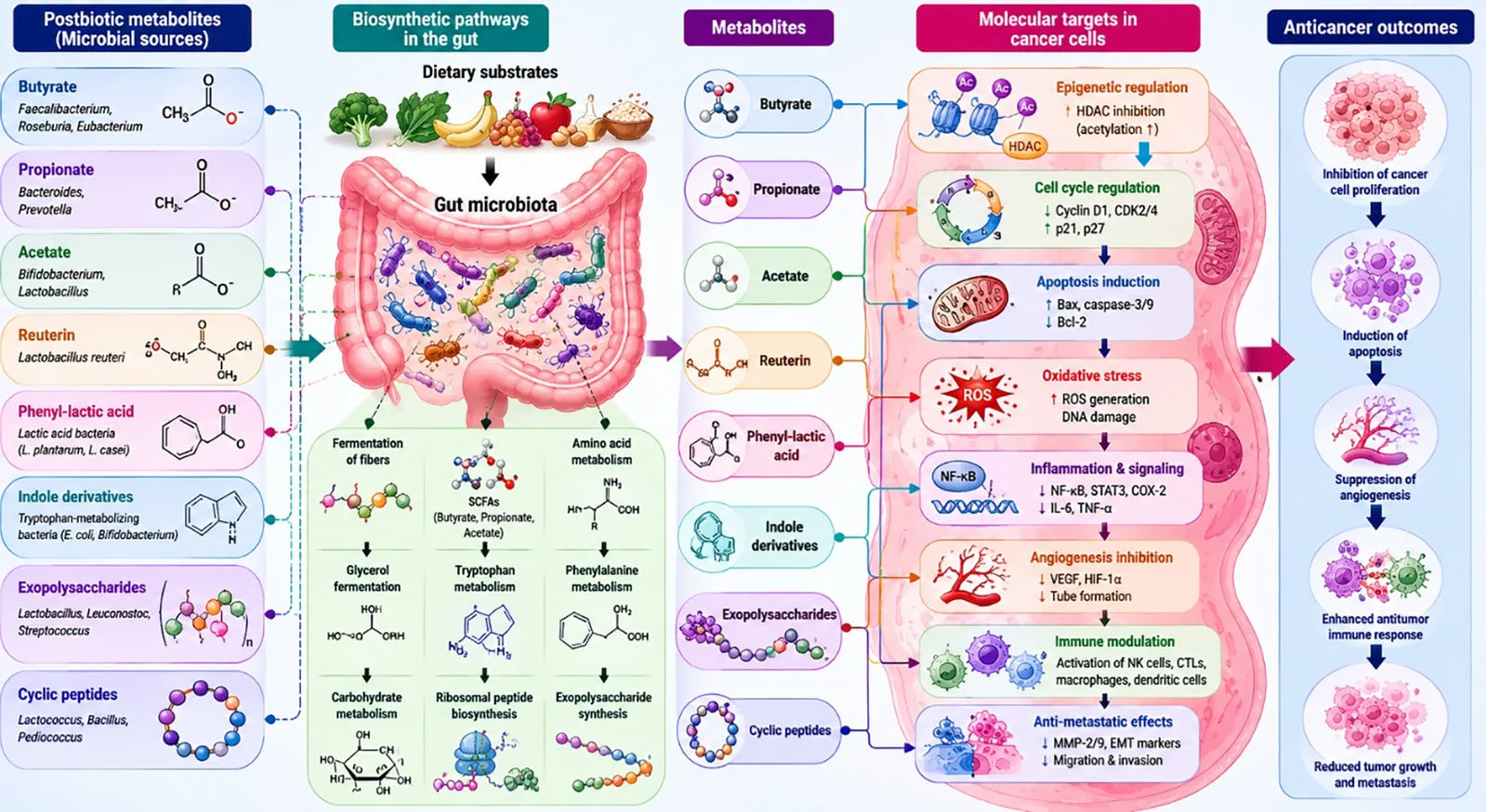
Microbial producers of anticancer bacteriocins.

Butyrate is one of the most extensively studied microbial metabolites because of its crucial roles in intestinal health and cancer biology (86). It is primarily produced through the fermentation of dietary fibre by gut bacteria belonging to the genera *Faecalibacterium*, *Roseburia*, and *Eubacterium* (86). As the principal energy source for colonocytes, butyrate contributes significantly to epithelial barrier maintenance and intestinal homeostasis (86). In cancer biology, butyrate functions as a histone deacetylase (HDAC) inhibitor, enabling epigenetic regulation of gene expression (**Figure 3**) (87). Through these mechanisms, butyrate influences cell-cycle regulation, apoptosis, cellular differentiation, and immune responses (87). Numerous studies have demonstrated that butyrate inhibits colorectal cancer cell proliferation, promotes programmed cell death, suppresses angiogenesis, and reduces inflammatory signalling (86, 87). Its ability to selectively target malignant cells while preserving normal epithelial function has made butyrate a promising candidate for microbiome-based cancer therapeutics (87).

## Propionate

Propionate is another important short-chain fatty acid (SCFA) generated through the microbial fermentation of complex carbohydrates (88). Although traditionally associated with metabolic regulation and glucose homeostasis, increasing evidence suggests that propionate also possesses anticancer properties (88). Similar to butyrate, propionate can inhibit histone deacetylase (HDAC) activity and modulate transcriptional programmes involved in tumour development (89). Experimental studies have demonstrated that propionate suppresses cancer cell proliferation, induces apoptosis, and enhances antitumour immune responses (89). Furthermore, its anti-inflammatory effects may contribute to cancer prevention and improve therapeutic efficacy (88, 89).

## Acetate

Acetate is the most abundant SCFA produced by the intestinal microbiota and serves as an important metabolic link between microbial and host physiology (84). While acetate supports cellular energy metabolism in both healthy and malignant tissues, emerging evidence indicates that its biological effects are highly context-dependent (84). Acetate participates in immune regulation, lipid metabolism, cellular signalling, and epigenetic processes (84, 87). Recent studies suggest that acetate may influence oxidative stress responses and tumour metabolism, thereby affecting cancer progression (87). Additionally, acetate-producing microorganisms contribute to microbiome stability, which may indirectly influence cancer susceptibility and treatment outcomes (84).

## Reuterin

Reuterin is a multifunctional antimicrobial metabolite produced during glycerol fermentation by *Limosilactobacillus reuteri* (65, 68). Chemically, reuterin consists of a dynamic mixture of aldehydes and related compounds with broad-spectrum biological activity (68). Beyond its antimicrobial effects, reuterin has attracted considerable attention because of its potential anticancer properties (69). Studies indicate that reuterin selectively induces oxidative stress in tumour cells, disrupts cellular redox homeostasis, and triggers apoptosis (88). The heightened sensitivity of cancer cells to oxidative imbalance may provide a therapeutic window that can be exploited for anticancer interventions (88).

## Phenyl-lactic acid

Phenyl-lactic acid is an aromatic organic acid produced by lactic acid bacteria through amino acid metabolism (66). Although initially recognized for its antimicrobial activity, growing evidence suggests that it possesses broader biological functions (66, 67). Experimental studies have demonstrated antioxidant, anti-inflammatory, and immunomodulatory activities that may contribute to the suppression of tumour-promoting microenvironments (67). While its direct anticancer mechanisms remain incompletely understood, phenyl-lactic acid represents a promising candidate for further investigation as a microbiome-derived therapeutic molecule (69).

## Indole derivatives

Indole derivatives constitute a diverse group of microbial metabolites generated through tryptophan metabolism (84). These compounds serve as important signalling molecules that mediate communication between the microbiota and host tissues (84). Many indole metabolites activate the aryl hydrocarbon receptor (AhR) and other regulatory pathways involved in immune function, epithelial barrier integrity, and inflammation (89). Dysregulation of tryptophan metabolism has been implicated in multiple cancers, and several indole derivatives have demonstrated antiproliferative, immunomodulatory, and antitumour activities (89). Consequently, these metabolites are increasingly being explored as potential agents for cancer prevention and immunotherapy (84, 89).

## Exopolysaccharides

Microbial exopolysaccharides are high-molecular-weight polymers secreted into the extracellular environment (64). These molecules contribute to biofilm formation, microbial adhesion, environmental adaptation, and host–microbe interactions (64, 65). Recent studies have demonstrated that exopolysaccharides possess antioxidant, anti-inflammatory, immunomodulatory, and anticancer properties (66). Their ability to stimulate macrophages, dendritic cells, natural killer cells, and cytotoxic lymphocytes has generated significant interest in oncology (67). Furthermore, their biocompatibility and structural versatility make them attractive candidates for drug delivery systems and nanomedicine applications (66, 67).

## Cyclic peptides

Cyclic peptides represent a structurally unique class of microbial metabolites characterized by the covalent linkage of peptide termini or side chains, resulting in highly stable molecular architectures (90). Cyclization enhances resistance to enzymatic degradation, improves receptor specificity, and often confers favourable pharmacokinetic properties (78, 90). A large number of cyclic peptides have demonstrated antimicrobial, anti-inflammatory, and anticancer activities (78). Their structural stability and ability to interact with intracellular targets make them particularly attractive candidates for drug discovery (78, 86). Several clinically successful therapeutics have been derived from cyclic peptide scaffolds, highlighting their substantial biomedical potential (**Table 2**) (86). In the context of microbiome-derived therapeutics, cyclic peptides provide a promising platform for the development of novel inhibitors targeting cancer-associated signalling pathways (86, 90).

**Table 2:** Major postbiotic metabolites and their anticancer activities.

Metabolite	Microbial source	Biosynthetic precursor	Major mechanisms	Anticancer effects	Drug discovery potential
Butyrate	Gut anaerobes	Dietary fiber	HDAC inhibition	Apoptosis, cell-cycle arrest	Very High
Propionate	Gut microbiota	Carbohydrate fermentation	Epigenetic regulation	Growth suppression	High
Acetate	Diverse bacteria	Carbohydrate metabolism	Metabolic regulation	Immune modulation	Moderate
Reuterin	*Lactobacillus reuteri*	Glycerol	Oxidative stress induction	Tumor cell death	High
Phenyl-lactic acid	LAB species	Phenylalanine	Antioxidant activity	Anti-inflammatory effects	Emerging
Indole derivatives	Tryptophan-metabolizing bacteria	Tryptophan	AhR signaling	Immune regulation	Very High
Exopolysaccharides	LAB and probiotics	Carbohydrates	Immune activation	Tumor suppression	High
Cyclic peptides	Various bacteria	Ribosomal peptides	Multiple molecular targets	Cytotoxic activity	Very High

## Pharmacological applications of postbiotic metabolites

The pharmacological significance of postbiotic metabolites extends far beyond their roles in microbial ecology and host–microbe interactions (88). These compounds represent a vast and largely untapped reservoir of structurally diverse bioactive molecules with the potential to modulate multiple therapeutic targets simultaneously (88). As naturally occurring products of microbial metabolism, many postbiotic metabolites exhibit favourable safety profiles and lower toxicity than conventional anticancer agents (89). Their multifunctional biological activities make them attractive candidates for use as standalone therapeutics or as adjuvants that enhance the efficacy of existing cancer treatments (88, 89).

A growing body of evidence indicates that postbiotic metabolites can regulate several fundamental processes involved in cancer initiation, progression, and metastasis (91). These include chronic inflammation, oxidative stress, immune dysregulation, epigenetic alterations, angiogenesis, cellular proliferation, and metastatic dissemination (91). By targeting multiple cancer-related pathways, postbiotic metabolites offer opportunities to overcome some of the limitations associated with conventional therapies, including drug resistance and off-target toxicity (91, 92). Furthermore, advances in metabolomics, computational chemistry, molecular docking, molecular dynamics simulations, and artificial intelligence (AI)-driven drug discovery have accelerated the identification and optimization of metabolite-derived lead compounds (92). As our understanding of microbiome–host interactions continues to expand, postbiotic metabolites are increasingly being recognized as valuable components of next-generation anticancer strategies (92, 93). Their integration into precision medicine approaches may ultimately facilitate the development of safer, more effective, and biologically informed interventions for cancer prevention and treatment (93).

## Cancer hallmarks targeted by bacteriocin-derived and postbiotic molecules

The concept of cancer hallmarks provides a comprehensive framework for understanding the biological capabilities acquired during tumour initiation, progression, and metastasis (15, 19). These hallmarks include sustained proliferative signalling, resistance to programmed cell death, induction of angiogenesis, invasion and metastasis, immune evasion, chronic inflammation, and metabolic reprogramming (19, 28). Because these processes are highly interconnected, they have become major targets in the development of modern anticancer therapeutics aimed at achieving durable clinical responses (28, 33, 45).

Recent studies have identified bacteriocins and postbiotic-derived small molecules as promising therapeutic candidates capable of modulating multiple cancer hallmarks simultaneously (33, 44). Unlike many conventional anticancer drugs that primarily target a single molecular pathway, microbiome-derived metabolites frequently exhibit pleiotropic effects across diverse cellular and molecular networks involved in tumour progression (44, 94). These compounds have been reported to influence cellular proliferation, apoptosis, oxidative stress, angiogenesis, immune responses, inflammatory pathways, and tumour–microenvironment interactions (44, 94). Such multifaceted mechanisms of action make them particularly attractive for combating the complexity and heterogeneity of cancer (3, 7, 11, 12).

Consequently, considerable research efforts are being directed toward elucidating the molecular mechanisms underlying the anticancer activities of bacteriocins and postbiotic-derived compounds (87). A deeper understanding of their interactions with cancer-associated pathways will facilitate the rational design of novel therapeutics and support the development of microbiome-inspired approaches for precision oncology (89).

Several experimental studies have demonstrated the pro-apoptotic activity of bacteriocins in various cancer models. Nisin, a lantibiotic produced by *Lactococcus lactis*, has been shown to inhibit the growth of head and neck squamous cell carcinoma by increasing intracellular calcium influx, activating CHAC1- and caspase-mediated apoptotic pathways, and significantly reducing tumour volume in xenograft mouse models (95, 96). In addition to inducing apoptosis, nisin suppresses tumour cell proliferation while exhibiting minimal cytotoxicity toward normal epithelial cells, highlighting its potential as a selective anticancer peptide (95, 96).

Similarly, pediocin PA-1, produced by *Pediococcus acidilactici*, has demonstrated selective cytotoxicity against HCT-15 (colorectal cancer) and HeLa (cervical cancer) cell lines (97). Mechanistic studies suggest that pediocin interacts with negatively charged phospholipids on the cancer cell membrane, resulting in membrane permeabilization, mitochondrial dysfunction, cytochrome *c* release, and activation of the intrinsic apoptotic pathway (97).

Among microbiome-derived postbiotic metabolites, butyrate has been extensively investigated in colorectal cancer models. Experimental evidence indicates that butyrate functions as a histone deacetylase (HDAC) inhibitor, leading to cell-cycle arrest, activation of caspase-3 and caspase-9, upregulation of the pro-apoptotic protein Bax, downregulation of the anti-apoptotic protein Bcl-2, and subsequent induction of apoptosis (98, 99). In addition, butyrate suppresses NF-κB signalling, reduces chronic inflammatory responses, and enhances antitumour immune activity, further contributing to its anticancer effects (98, 99).

Another promising microbiome-derived metabolite is reuterin, produced by *Limosilactobacillus reuteri*. Recent studies have shown that reuterin selectively inhibits the growth of colorectal cancer cells by disrupting intracellular redox homeostasis, promoting excessive reactive oxygen species (ROS) generation, and inducing ROS-mediated apoptosis (100). Collectively, these findings demonstrate that bacteriocins and microbiome-derived postbiotic metabolites induce apoptosis through multiple complementary mechanisms, including membrane disruption, mitochondrial dysfunction, epigenetic regulation, oxidative stress, and activation of caspase-dependent signalling pathways. Their ability to selectively target malignant cells while sparing normal tissues highlights their considerable potential as promising candidates for the development of next-generation microbiome-based anticancer therapeutics.

## Targeting sustained proliferative signalling

Sustained proliferative signalling is a fundamental hallmark of cancer that enables malignant cells to continuously grow and divide despite normal regulatory constraints (89). This phenotype is commonly driven by oncogenic mutations, dysregulation of growth factor receptors, and persistent activation of signalling pathways such as PI3K/Akt/mTOR, MAPK/ERK, Wnt/β-catenin, and JAK/STAT (89, 91). Continuous stimulation of these pathways allows tumour cells to evade normal growth-control mechanisms and maintain uncontrolled proliferation (91).

Numerous bacteriocins and postbiotic metabolites have demonstrated the ability to suppress proliferative signalling (92). Nisin, one of the most extensively studied lantibiotics, inhibits cancer cell proliferation through disruption of membrane integrity and alterations in intracellular calcium homeostasis (92). Similarly, plantaricins, pediocins, and enterocins have exhibited growth-inhibitory effects in multiple cancer models (92, 93). Postbiotic metabolites such as butyrate and propionate exert antiproliferative activity through epigenetic mechanisms, particularly by inhibiting histone deacetylases (HDACs) (93). These effects alter transcriptional programmes involved in cyclin expression, cell-cycle regulation, and cellular differentiation (93). Furthermore, microbiome-derived molecules have been shown to suppress key oncogenic pathways by inhibiting mTOR activation, reducing β-catenin nuclear translocation, and attenuating PI3K/Akt signalling (93). The ability of these compounds to simultaneously target multiple growth-regulatory pathways may provide therapeutic advantages over single-target interventions and potentially reduce the development of drug resistance (92, 93).

## Overcoming resistance to apoptosis

Evasion of programmed cell death represents a critical adaptation that enables cancer cells to survive despite genetic instability, metabolic stress, and therapeutic intervention (95). Resistance to apoptosis is frequently associated with overexpression of anti-apoptotic proteins such as Bcl-2 and survivin, loss of tumour suppressor function, and disruption of mitochondrial signalling pathways (95). Increasing evidence suggests that bacteriocins possess intrinsic pro-apoptotic activity (96). Several bacteriocins interact directly with cancer cell membranes, causing membrane permeabilization and disruption of ionic homeostasis (96). These events initiate intracellular stress responses that ultimately trigger apoptosis (96). Experimental studies have demonstrated activation of caspase-dependent pathways, increased expression of pro-apoptotic proteins such as Bax, release of cytochrome c from mitochondria, and downregulation of anti-apoptotic proteins following bacteriocin treatment (84, 95, 96).

**Figure 4:**
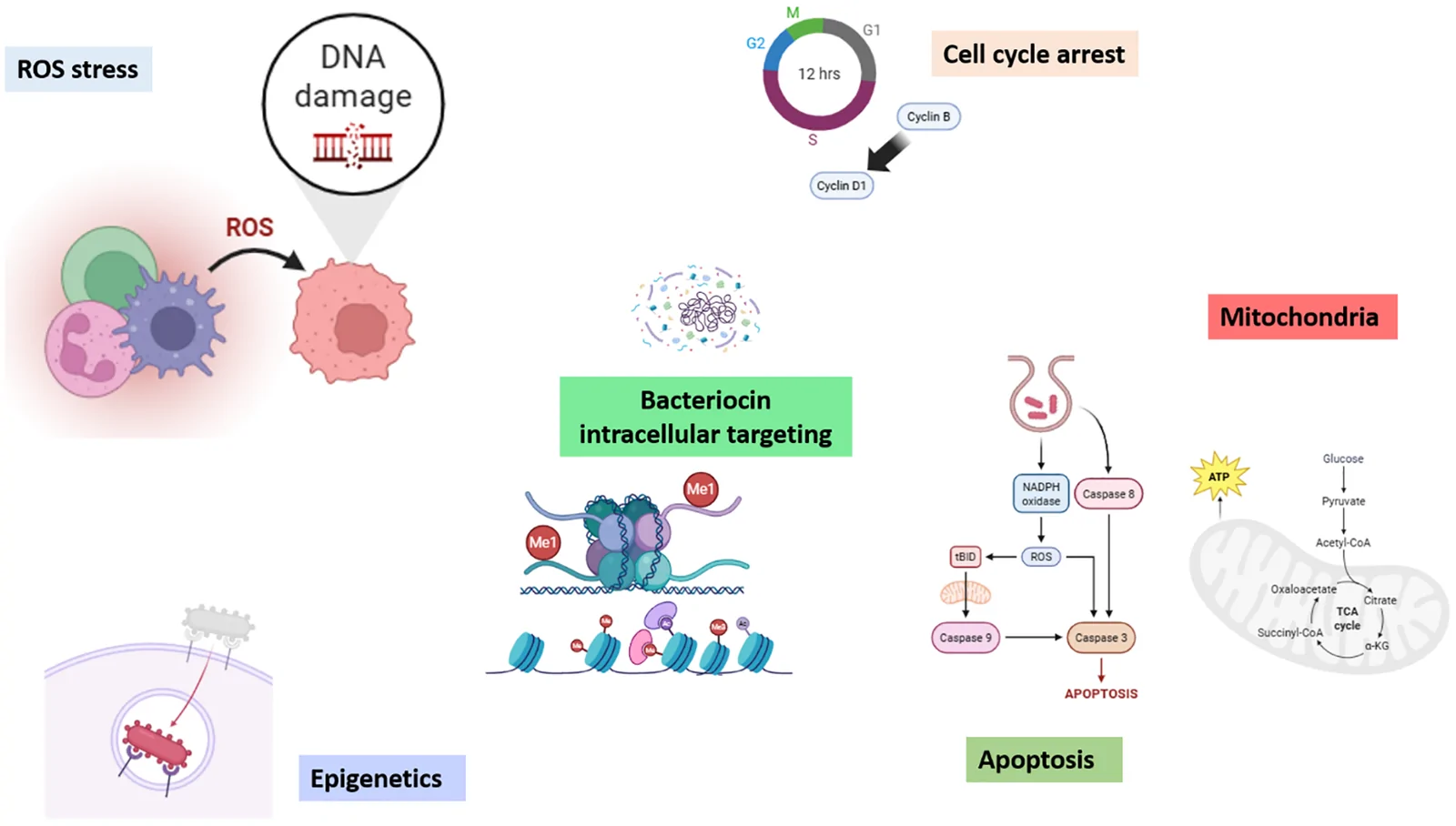
Intracellular mechanisms of anticancer activity.

Postbiotic metabolites also promote apoptosis through multiple complementary mechanisms. Among these metabolites, butyrate is one of the best-characterized compounds and functions as a histone deacetylase (HDAC) inhibitor, resulting in epigenetic modulation of gene expression. HDAC inhibition upregulates pro-apoptotic genes such as Bax and p21, suppresses anti-apoptotic proteins including Bcl-2, activates caspase-3 and caspase-9, and induces cell-cycle arrest, ultimately promoting apoptosis in colorectal and other cancer cells (**Figure 4**) (98, 99). Reuterin, produced by *Limosilactobacillus reuteri*, exerts anticancer activity by disrupting intracellular redox homeostasis, leading to excessive reactive oxygen species (ROS) generation, mitochondrial dysfunction, DNA damage, and activation of intrinsic apoptotic pathways (100). Because cancer cells generally exhibit elevated basal oxidative stress compared with normal cells, they are more susceptible to reuterin-induced ROS accumulation, providing a degree of therapeutic selectivity (100). Restoration of apoptotic sensitivity is particularly important in drug-resistant tumours, where dysregulation of cell-death pathways contributes to treatment failure. Consequently, microbiome-derived postbiotic metabolites have emerged as promising therapeutic candidates capable of overcoming apoptosis resistance while enhancing the efficacy of conventional anticancer therapies (67, 101, 102).

## Inhibition of tumour angiogenesis

For tumours to grow beyond a limited size, they require a continuous supply of oxygen and nutrients through the formation of new blood vessels, a process known as angiogenesis. This process is primarily regulated by pro-angiogenic mediators, including vascular endothelial growth factor (VEGF), fibroblast growth factors (FGFs), and hypoxia-inducible factor-1α (HIF-1α), which stimulate endothelial cell proliferation, migration, and neovascularization (98). Enhanced angiogenesis not only sustains tumour growth but also facilitates metastatic dissemination by providing tumour cells with access to the circulation (98).

Emerging evidence indicates that several microbiome-derived metabolites possess anti-angiogenic activity. Butyrate has been shown to suppress VEGF expression, inhibit endothelial cell proliferation, and reduce microvessel formation through inhibition of histone deacetylases (HDACs) and modulation of HIF-1α and NF-κB signalling pathways (98, 100). Experimental studies have further demonstrated that butyrate decreases the production of pro-inflammatory cytokines, including IL-6 and TNF-α, thereby reducing the inflammatory microenvironment that promotes angiogenesis (100).

In addition, bacteriocins may indirectly inhibit angiogenesis by suppressing inflammatory signalling pathways and reducing the secretion of pro-angiogenic mediators (100). Several cyclic peptides and other microbiome-derived metabolites have also been reported to interfere with endothelial cell migration, tube formation, and extracellular matrix remodelling, thereby impairing new blood vessel formation (98, 100). Although most evidence is currently derived from *in vitro* and preclinical studies, these findings suggest that bacteriocins and postbiotic metabolites could serve as promising adjuncts to existing anti-angiogenic therapies.

## Inhibition of tumour invasion and metastasis

Metastasis is responsible for approximately 90% of cancer-related deaths and remains one of the greatest challenges in oncology (99). The metastatic cascade involves epithelial–mesenchymal transition (EMT), degradation of the extracellular matrix (ECM), tumour cell migration, intravasation into the circulation, extravasation, and colonization of distant organs (99).

Several bacteriocins have demonstrated the ability to suppress metastatic progression by modulating signalling pathways associated with EMT, cell adhesion, and extracellular matrix remodelling. Experimental studies have reported that bacteriocin treatment downregulates matrix metalloproteinases (MMP-2 and MMP-9), preserves E-cadherin expression, suppresses N-cadherin and vimentin, and consequently reduces cancer cell migration and invasion (97, 99). Among postbiotic metabolites, butyrate has been shown to inhibit EMT by regulating transcription factors such as Snail, Slug, and ZEB1, while restoring epithelial characteristics in malignant cells (96). Indole derivatives, generated through microbial tryptophan metabolism, activate the aryl hydrocarbon receptor (AhR) and modulate signalling pathways involved in cell migration, epithelial integrity, and immune regulation, thereby reducing metastatic potential (85). Furthermore, microbiome-derived metabolites influence interactions between tumour cells and stromal, immune, and endothelial cells within the tumour microenvironment, creating conditions that are less favourable for tumour dissemination (55, 67, 71). Collectively, these findings indicate that bacteriocins and postbiotic metabolites inhibit tumour invasion and metastasis by targeting multiple stages of the metastatic cascade, suggesting their potential as complementary agents in anti-metastatic cancer therapy.

## Modulation of immune escape mechanisms

Cancer cells evade immune surveillance through multiple mechanisms, including downregulation of antigen presentation, upregulation of immune checkpoint molecules such as PD-L1, recruitment of regulatory T cells (Tregs) and myeloid-derived suppressor cells (MDSCs), and secretion of immunosuppressive cytokines including IL-10 and TGF-β (103). These processes establish an immunosuppressive tumour microenvironment that promotes tumour progression and reduces the effectiveness of anticancer therapies (103).

Recent evidence demonstrates that the gut microbiome plays a central role in regulating both innate and adaptive immunity, with microbiome-derived metabolites acting as key mediators of host immune responses (104). Short-chain fatty acids (SCFAs), particularly butyrate, propionate, and acetate, regulate immune cell differentiation through epigenetic mechanisms, promote dendritic cell maturation, enhance macrophage function, and stimulate the activation of natural killer (NK) cells and CD8^+^ cytotoxic T lymphocytes, thereby strengthening antitumour immunity (104, 105).

Bacteriocins may also contribute to immune regulation through both direct and indirect mechanisms. Experimental studies suggest that certain bacteriocins stimulate cytokine production, enhance antigen presentation, and promote immune-cell activation while simultaneously modulating gut microbial composition to establish a more favourable immunological environment (103). Furthermore, microbiome-derived metabolites have been reported to improve responses to immune checkpoint inhibitors by enhancing T-cell infiltration into tumours and reducing chronic inflammation, highlighting their potential as adjuvants for cancer immunotherapy (104, 105). Although most mechanistic evidence has been generated in preclinical models, these findings collectively suggest that bacteriocins and postbiotic metabolites can overcome tumour-induced immune suppression and may complement existing immunotherapeutic strategies by enhancing endogenous antitumour immune responses.

## Suppression of cancer-associated inflammation

Chronic inflammation is widely recognized as a hallmark of cancer and contributes to tumour initiation, progression, metastasis, and therapeutic resistance (106). Persistent inflammatory signalling promotes genomic instability, DNA damage, uncontrolled cellular proliferation, angiogenesis, and immune dysregulation, thereby creating a tumour-promoting microenvironment (106, 107). Key inflammatory pathways involved in carcinogenesis include NF-κB, STAT3, cyclooxygenase-2 (COX-2), tumour necrosis factor-α (TNF-α), and interleukin-6 (IL-6), all of which regulate genes associated with cell survival, proliferation, angiogenesis, and metastasis (107).

Several microbiome-derived postbiotic metabolites exhibit potent anti-inflammatory activity by modulating these signalling pathways. Butyrate, propionate, and acetate suppress NF-κB activation, reduce the production of pro-inflammatory cytokines such as TNF-α, IL-1β, and IL-6, and inhibit COX-2 expression, thereby attenuating chronic inflammatory signalling associated with tumour progression (106, 107). Among these metabolites, butyrate has been extensively investigated because it functions as both a histone deacetylase (HDAC) inhibitor and an immunomodulatory molecule, enabling simultaneous epigenetic regulation and suppression of inflammatory gene expression (107, 108).

Indole derivatives, including indole-3-propionic acid and indole-3-aldehyde, contribute to immune homeostasis through activation of the aryl hydrocarbon receptor (AhR), which regulates intestinal barrier integrity, mucosal immunity, and inflammatory responses (108). Appropriate AhR signalling limits chronic inflammation and may reduce the risk of inflammation-associated tumour development (108).

In addition, bacteriocins may indirectly reduce inflammation by selectively inhibiting pathogenic microorganisms associated with microbial dysbiosis, thereby restoring microbial homeostasis and decreasing the production of inflammatory mediators (106). Although most evidence remains preclinical, the combined anti-inflammatory and immunomodulatory properties of bacteriocins and postbiotic metabolites highlight their considerable potential as adjunctive therapeutic agents for preventing inflammation-driven carcinogenesis and enhancing anticancer treatment efficacy.

## Targeting metabolic reprogramming

Metabolic reprogramming is a defining characteristic of cancer that enables malignant cells to adapt to nutrient deprivation and sustain rapid proliferation (90). One of the best-characterized metabolic alterations is the Warburg effect, in which tumour cells preferentially utilize aerobic glycolysis despite the presence of oxygen (90). Cancer cells also undergo profound alterations in amino acid metabolism, lipid biosynthesis, mitochondrial function, and redox homeostasis to support tumour growth and survival (94).

Microbiome-derived metabolites have emerged as important regulators of these metabolic pathways. Short-chain fatty acids (SCFAs) activate AMP-activated protein kinase (AMPK) while suppressing mechanistic target of rapamycin (mTOR) signalling, thereby inhibiting anabolic metabolism, reducing cellular proliferation, and promoting apoptosis (86). Butyrate additionally functions as a histone deacetylase (HDAC) inhibitor, providing a mechanistic link between cellular metabolism, epigenetic regulation, and gene expression (86).

Other microbiome-derived metabolites exert distinct metabolic effects. Reuterin disrupts intracellular redox homeostasis by increasing reactive oxygen species (ROS), leading to mitochondrial dysfunction and oxidative stress-mediated apoptosis in cancer cells (94). Indole derivatives regulate tryptophan metabolism through AhR-dependent signalling, thereby influencing both metabolic adaptation and immune responses within the tumour microenvironment (86). Several bacteriocins have also been reported to impair mitochondrial membrane integrity, reduce ATP production, and interfere with cellular bioenergetics, ultimately limiting the metabolic flexibility required for tumour progression (41, 49).

Collectively, these findings indicate that microbiome-derived metabolites target multiple metabolic vulnerabilities in cancer cells. Their ability to simultaneously regulate energy metabolism, epigenetic signalling, oxidative stress, and mitochondrial function provides a strong rationale for developing microbiome-based metabolic therapies either alone or in combination with conventional anticancer agents.

## Integrative targeting of cancer hallmarks

One of the most distinctive characteristics of bacteriocins and postbiotic metabolites is their ability to simultaneously regulate multiple hallmarks of cancer. Unlike conventional anticancer drugs that frequently target a single molecular pathway, microbiome-derived compounds influence interconnected biological networks controlling cell proliferation, apoptosis, inflammation, immune surveillance, angiogenesis, metabolic reprogramming, invasion, and metastasis (3, 5). This multitarget mode of action may reduce the likelihood of therapeutic resistance while enhancing overall treatment efficacy (6, 9).

Recent advances in molecular docking, virtual screening, molecular dynamics simulations, network pharmacology, systems biology, and artificial intelligence (AI) have accelerated the identification and optimization of microbiome-derived bioactive compounds with anticancer potential (13). These computational approaches enable prediction of target–ligand interactions, assessment of binding stability, identification of multitarget compounds, and optimization of pharmacokinetic and toxicity profiles before experimental validation (19). Furthermore, integration of multi-omics datasets, including metagenomics, metabolomics, transcriptomics, and proteomics, has improved understanding of host–microbiome interactions and facilitated the discovery of novel therapeutic targets for precision oncology (19).

Despite these promising advances, most microbiome-derived anticancer agents remain at the preclinical stage, and additional mechanistic studies, standardized experimental protocols, and well-designed clinical trials are required to establish their safety, efficacy, pharmacokinetics, and therapeutic applicability in humans. Nevertheless, the integration of microbiome science with computational drug discovery and precision medicine provides a promising framework for developing next-generation multitarget anticancer therapeutics capable of addressing the biological complexity and heterogeneity of cancer (3, 5, 6, 9, 13, 19).

## Artificial intelligence in bacteriocin-based anticancer drug discovery

Artificial intelligence (AI) is transforming cancer drug discovery by accelerating the identification, optimization, and development of novel therapeutic agents (44, 55). The rapid expansion of genomic, proteomic, metabolomic, and structural databases has generated vast amounts of biological data that exceed the analytical capacity of conventional computational methods (48). AI-based approaches integrate these multidimensional datasets to identify novel therapeutic targets, predict drug–target interactions, optimize lead compounds, and prioritize candidates for experimental validation, thereby reducing both the time and cost associated with traditional drug discovery pipelines (55, 71).

In microbiome-based drug discovery, machine learning (ML) algorithms have been successfully applied to predict the biological activity, antimicrobial potential, anticancer efficacy, pharmacokinetic properties, and toxicity profiles of bacteriocins and other microbial peptides using peptide sequences, molecular descriptors, and physicochemical characteristics (44, 48). Supervised learning models, including random forest, support vector machine (SVM), and XGBoost, have demonstrated high predictive accuracy for classifying antimicrobial and anticancer peptides, while unsupervised learning approaches have facilitated the identification of structure–activity relationships and functional peptide families (55, 71). These computational strategies enable the rapid screening of thousands of naturally occurring and synthetic peptide sequences before laboratory validation, substantially improving screening efficiency.

Deep learning (DL) has further enhanced computational drug discovery by identifying complex nonlinear relationships within large biological datasets that cannot be detected using conventional statistical approaches (83). Convolutional neural networks (CNNs), recurrent neural networks (RNNs), and transformer-based architectures have been employed to predict peptide function, drug–target interactions, protein–protein interactions, toxicity, and biomarker signatures relevant to cancer therapy (93). Recent studies have demonstrated that deep-learning models outperform traditional machine-learning algorithms in predicting anticancer peptide activity, enabling more accurate prioritization of lead molecules for experimental investigation (95).

The emergence of generative artificial intelligence has created new opportunities for *de novo* peptide design. Generative models, including variational autoencoders (VAEs), generative adversarial networks (GANs), and transformer-based language models, can design entirely new peptide sequences with predefined biological properties, including enhanced stability, improved target specificity, reduced toxicity, and increased anticancer activity (96). These approaches explore chemical and sequence spaces that are inaccessible through conventional screening methods and therefore accelerate the optimization of bacteriocin-derived therapeutic candidates.

Recent advances in protein structure prediction have further strengthened AI-assisted drug discovery. AlphaFold has revolutionized structural biology by accurately predicting three-dimensional protein structures directly from amino acid sequences, thereby facilitating structure-based drug design, binding-site prediction, molecular docking, and mechanistic studies of bacteriocin–protein interactions (103). The availability of high-confidence structural models enables researchers to investigate peptide–receptor interactions, optimize binding affinity, and design structurally improved bacteriocin analogues with enhanced therapeutic potential (104, 105).

AI-assisted molecular docking and molecular dynamics (MD) simulations have become indispensable tools for evaluating microbiome-derived bioactive compounds against cancer-associated molecular targets. Machine learning-enhanced docking algorithms improve ligand-pose prediction, binding-affinity estimation, and virtual-screening accuracy compared with conventional docking approaches (93). Subsequent molecular dynamics simulations assess the stability of protein–ligand complexes under physiological conditions, providing valuable insights into conformational changes, interaction stability, and binding mechanisms before experimental validation (83, 95). These integrated computational workflows have been successfully employed to identify promising bacteriocins and postbiotic metabolites targeting proteins involved in apoptosis, angiogenesis, cell-cycle regulation, and tumour progression.

Beyond individual drug candidates, AI has also facilitated the integration of multi-omics datasets, including metagenomics, transcriptomics, proteomics, metabolomics, and microbiome profiling, to identify novel therapeutic targets and predictive biomarkers for precision oncology (105). Network pharmacology combined with AI enables systematic analysis of complex host–microbiome interactions and predicts multitarget therapeutic mechanisms, which is particularly important because microbiome-derived metabolites frequently regulate multiple signalling pathways simultaneously (66).

Despite these remarkable advances, several challenges remain before AI-guided bacteriocin discovery can be translated into clinical practice. Most predictive models rely on relatively small and heterogeneous datasets, limiting model generalizability and increasing the risk of algorithmic bias (93). Furthermore, experimental validation remains essential to confirm AI-generated predictions, and standardized datasets, transparent model development, and interpretable algorithms are needed to improve reproducibility and regulatory acceptance (83, 96).

Overall, the convergence of artificial intelligence, deep learning, AlphaFold-based structural prediction, molecular docking, molecular dynamics simulations, network pharmacology, and multi-omics analysis is transforming microbiome-based anticancer drug discovery. These computational technologies are expected to accelerate the identification and optimization of bacteriocins and postbiotic-derived therapeutics, thereby supporting the development of safer, more effective, and highly personalized anticancer strategies for precision oncology (66, 83, 93, 95, 96, 103–105).

## Nanotechnology, challenges, and future perspectives in gut microbiota-derived bioactive metabolites for cancer therapy

### Nanotechnology and targeted drug delivery

The therapeutic potential of gut microbiota-derived bioactive metabolites has generated considerable interest in the development of advanced drug delivery systems designed to overcome their pharmacological limitations (79, 82). Although metabolites such as bacteriocins, short-chain fatty acids (SCFAs), exopolysaccharides, indole derivatives, reuterin, and other postbiotic molecules exhibit promising anticancer activities, their clinical application is often limited by poor physicochemical stability, rapid degradation in the gastrointestinal tract and systemic circulation, low bioavailability, short half-life, and insufficient accumulation within tumour tissues (79, 89). To address these limitations, nanotechnology-based drug delivery platforms have emerged as promising strategies for improving the stability, controlled release, targeted delivery, and therapeutic efficacy of microbiome-derived bioactive compounds (82, 95).

Among the various nanocarrier systems, lipid nanoparticles (LNPs) have attracted considerable attention because of their excellent biocompatibility, biodegradability, and ability to encapsulate both hydrophilic and hydrophobic microbial metabolites (95, 97). LNPs protect sensitive bioactive molecules from enzymatic degradation, facilitate efficient intracellular delivery, and prolong systemic circulation (95). Surface modification with polyethylene glycol (PEG) or tumour-targeting ligands further enhances circulation time and promotes both passive and active accumulation within tumour tissues (97). Recent studies have demonstrated that lipid-based nanocarriers significantly improve the stability and therapeutic efficacy of antimicrobial peptides and microbiome-derived bioactive molecules in preclinical cancer models (95, 97).

Polymeric nanoparticles, fabricated from biodegradable materials such as poly(lactic-co-glycolic acid) (PLGA), chitosan, alginate, and polyethylene glycol (PEG), represent another extensively investigated drug delivery platform (82, 95). These nanoparticles provide sustained and controlled drug release, improve pharmacokinetic properties, and protect encapsulated metabolites from premature degradation (82). Furthermore, their surfaces can be functionalized with antibodies, peptides, aptamers, or small-molecule ligands to achieve receptor-mediated tumour targeting, thereby reducing off-target toxicity while enhancing therapeutic efficacy (97).

Liposomes remain among the most extensively studied nanocarriers for peptide-based therapeutics (95). Their phospholipid bilayer structure enables efficient encapsulation of microbiome-derived peptides and postbiotic compounds while minimizing systemic toxicity (95). Liposomal formulations exhibit improved stability, prolonged circulation, and enhanced tumour accumulation through the enhanced permeability and retention (EPR) effect (97). In addition, conjugation with targeting ligands enables selective delivery to cancer cells that overexpress specific surface receptors, thereby improving therapeutic specificity (97).

Hydrogel-based delivery systems have also attracted significant attention because they enable localized and sustained release of therapeutic agents (82). Injectable and stimuli-responsive hydrogels provide prolonged retention of microbiome-derived metabolites at tumour sites while minimizing systemic exposure (82). These platforms are particularly attractive for postoperative cancer management, localized chemotherapy, and tissue engineering applications where controlled drug release is required (82).

Another emerging strategy involves the use of extracellular vesicle (EV)-based delivery systems (89). Naturally secreted bacterial and mammalian extracellular vesicles possess excellent biocompatibility and intrinsic cell-targeting capabilities (89). They can transport proteins, peptides, nucleic acids, and microbial metabolites across biological barriers while protecting these cargo molecules from enzymatic degradation (89). Recent studies suggest that engineered EVs may serve as efficient nanocarriers for microbiome-derived therapeutics, offering improved delivery efficiency and lower immunogenicity than many synthetic nanocarrier systems (89).

Microencapsulation technologies have also gained considerable attention for protecting probiotic-derived metabolites during storage, gastrointestinal transit, and systemic administration (79). Biopolymers such as alginate, pectin, carrageenan, and chitosan are widely used as encapsulating materials because they improve metabolite stability while enabling controlled release under physiological conditions (79). These technologies are particularly valuable for the oral delivery of postbiotic formulations and functional foods intended for cancer prevention and supportive therapy (79).

In addition to conventional nanocarriers, stimuli-responsive nanoparticles have emerged as intelligent drug delivery systems capable of responding to changes in pH, redox potential, enzymatic activity, or temperature (95, 97). Because the tumour microenvironment differs substantially from normal tissues, these “smart” nanocarriers enable site-specific drug release while minimizing systemic toxicity (97). Furthermore, integrating nanotechnology with molecular imaging and theranostic approaches offers opportunities for simultaneous diagnosis, targeted therapy, and real-time monitoring of treatment responses (95).

Despite these significant advances, several challenges continue to limit the clinical translation of nanotechnology-assisted microbiome therapeutics (4, 11, 33). Most delivery systems remain at the preclinical stage, and comprehensive data regarding long-term safety, immunogenicity, pharmacokinetics, biodistribution, and biodegradation are still limited (82, 95). In addition, manufacturing complexity, large-scale reproducibility, formulation stability, regulatory approval, and production costs represent substantial barriers to commercialization (95, 97). The inherent variability in microbiome-derived metabolites, together with the absence of standardized purification, formulation, and quality-control protocols, further complicates therapeutic development (79, 82). Addressing these challenges will require interdisciplinary research, standardized manufacturing practices, and well-designed clinical studies to facilitate the successful translation of microbiome-derived nanotherapeutics into precision oncology (82, 95, 97, 105).

## Future perspectives

The future of bacteriocin-based anticancer therapeutics is closely aligned with advances in precision medicine, synthetic biology, nanotechnology, and computational sciences (105, 107–109). Precision oncology enables patient-specific treatment strategies based on molecular and genetic tumour profiling, and bacteriocins may contribute to this paradigm by targeting specific oncogenic pathways (110). Engineered probiotic systems represent an emerging strategy for *in situ* production and delivery of therapeutic bacteriocins directly within the tumour microenvironment, thereby enhancing efficacy while minimizing systemic toxicity (69, 80, 111). Synthetic biology further enables the rational design and optimization of bacteriocins with improved stability, specificity, and anticancer activity (105, 107). The integration of multi-omics technologies—including genomics, transcriptomics, proteomics, metabolomics, and microbiomics—provides a comprehensive framework for understanding tumour biology and host–microbiome interactions (108). These approaches facilitate the identification of novel therapeutic targets and predictive biomarkers (108). Nanotechnology complements these advances by addressing key pharmacokinetic limitations, including poor stability, low bioavailability, and inadequate tumour targeting (82, 95, 97). Collectively, these interdisciplinary innovations provide a strong foundation for the development of microbiome-derived anticancer therapeutics (105, 107, 112).

Despite these promising developments, several challenges remain, including limited clinical validation, lack of standardized protocols, manufacturing limitations, and unresolved regulatory frameworks (113). In addition, immunogenicity, resistance potential, and long-term safety require further investigation (114–117). Overall, the convergence of computational biology, synthetic biology, microbiome research, and advanced drug delivery systems is rapidly advancing the field (105, 107, 108). With continued mechanistic insights and rigorous clinical validation, bacteriocin-derived compounds hold strong potential to emerge as next-generation precision oncology therapeutics (118–120).

## Conclusion

Bacteriocins and bacteriocin-derived small molecules, together with other postbiotic metabolites originating from the microbiome, constitute an emerging and promising class of anticancer agents. Evidence from experimental, *in vivo*, and computational studies indicates that these molecules exert broad-spectrum anticancer effects through multiple complementary mechanisms, including disruption of cancer cell membranes, induction of apoptosis, regulation of oxidative stress, cell-cycle arrest, inhibition of angiogenesis and metastasis, modulation of epigenetic processes, and activation of immune responses. Unlike conventional chemotherapeutic agents that generally target a single molecular pathway, bacteriocin-based compounds act on several cancer hallmarks simultaneously, which may help reduce the likelihood of therapeutic resistance and improve treatment outcomes. Recent advances in computational approaches such as molecular docking, molecular dynamics simulations, systems biology, and artificial intelligence have significantly accelerated the identification and optimization of potential bacteriocin-derived anticancer candidates. In parallel, nanotechnology-based delivery systems have addressed several key limitations, including poor stability, low bioavailability, and inadequate tumour targeting, thereby improving their translational potential. However, despite these promising developments, the field is still in an early stage of clinical translation. The absence of standardized experimental protocols, limited *in vivo* and clinical studies, incomplete pharmacokinetic and toxicity data, and unresolved regulatory challenges remain major barriers. Additional concerns related to large-scale production, formulation stability, immunogenicity, and long-term safety also need to be carefully addressed before clinical application.

Bacteriocin-based therapeutics, supported by innovations in synthetic biology, nanomedicine, microbiome research, and artificial intelligence, represent a strong foundation for the development of next-generation anticancer strategies. With continued mechanistic studies, rigorous preclinical validation, and well-designed clinical trials, these microbiome-derived molecules may evolve into safe and effective components of future precision oncology approaches. Importantly, advancing bacteriocin-based therapeutics aligns with the United Nations Sustainable Development Goals (SDGs), particularly Good Health and Well-being by supporting the development of innovative and potentially safer cancer therapies, Industry, Innovation and Infrastructure through the integration of biotechnology, artificial intelligence, synthetic biology, and nanomedicine, and Responsible Consumption and Production by encouraging sustainable and biologically derived therapeutic platforms. Collectively, integrating microbiome-based drug discovery with emerging technological innovations could contribute to more accessible, precise, and sustainable cancer treatment strategies and support long-term improvements in global health and well-being.
